# *Wolbachia* Effect on *Drosophila melanogaster* Lipid and Carbohydrate Metabolism

**DOI:** 10.3390/insects14040357

**Published:** 2023-04-03

**Authors:** Evgenia K. Karpova, Margarita A. Bobrovskikh, Maksim A. Deryuzhenko, Olga D. Shishkina, Nataly E. Gruntenko

**Affiliations:** Institute of Cytology and Genetics SB RAS, 630090 Novosibirsk, Russia

**Keywords:** *Drosophila melanogaster*, carbohydrate and lipid metabolism, triglycerides, trehalose, glucose, food consumption, *tps1*, *treh*, *bmm*, *dInR*

## Abstract

**Simple Summary:**

A study to investigate the effect that different *Wolbachia pipientis* strains have on the carbohydrate and lipid metabolism, starvation resistance and feeding behavior of *Drosophila melanogaster* females has been performed. The data obtained demonstrate that *Wolbachia* most likely change their hosts’ metabolism in different aspects, including the effect on energy metabolism, fitness and behavior, to ensure the host’s competitive advantage over uninfected insects, which contributes to the spread of the bacteria in the population.

**Abstract:**

The effect of maternally inherited endosymbiotic bacteria *Wolbachia* on triglyceride and carbohydrate metabolism, starvation resistance and feeding behavior of *Drosophila melanogaster* females was studied. Eight *D. melanogaster* lines of the same nuclear background were investigated; one had no infection and served as the control, and seven others were infected with different *Wolbachia* strains pertaining to wMel and wMelCS groups of genotypes. Most of the infected lines had a higher overall lipid content and triglyceride level than the control line and their expression of the *bmm* gene regulating triglyceride catabolism was reduced. The glucose content was higher in the infected lines compared to that in the control, while their trehalose levels were similar. It was also found that the *Wolbachia* infection reduced the level of *tps1* gene expression (coding for enzyme for trehalose synthesis from glucose) and had no effect on *treh* gene expression (coding for trehalose degradation enzyme). The infected lines exhibited lower appetite but higher survival under starvation compared to the control. The data obtained may indicate that *Wolbachia* foster their hosts’ energy exchange through increasing its lipid storage and glucose content to ensure the host’s competitive advantage over uninfected individuals. The scheme of carbohydrate and lipid metabolism regulation under *Wolbachia’s* influence was suggested.

## 1. Introduction

Intracellular bacteria *Wolbachia pipientis* are the most wide-spread prokaryotic symbiont in invertebrates. Being maternally inherited, they can control the biological, morphological and behavioral features of their hosts. Thus, *Wolbachia*-caused fertility disorders such as cytoplasmic incompatibility, feminization, induced parthenogenesis and male-killing have been described in different insect species [1]. At the same time, infected hosts have been known to obtain a competitive advantage over uninfected individuals [2]. Today, more than 470 different strains of *Wolbachia* have been described [3] that are divided into 17 possible phylogenetic supergroups [4]. Some of these strains have been shown to provide their hosts with important cofactors [5,6,7], improving the host’s fertility [8,9,10,11] and protecting it from lethal RNA viruses [12,13,14,15,16]. However, if *Wolbachia’s* parasitic effects modulating the host’s fertility and hence increasing the bacteria’s spread in insect population have been sufficiently described, *Wolbachia’s* effect on their host’s survivability remains understudied. 

In all living beings, carbohydrates, proteins and lipids are the three main energy sources to sustain their vital function. Among them, lipids are the main way to store energy [17]. They also play a crucial role in many cell functions such as membrane formation, energy generation, intra-and intercellular signal transmission and cell death regulation [17]. Carbohydrates are another necessity for every living organism and are essential for growth, fertility and vitality. Two main carbohydrates circulating in *D. melanogaster* hemolymph are glucose and trehalose [18]. Trehalose is the most widespread carbohydrate circulating in insects [18]. It is a disaccharide consisting of two α-glucose molecules linked in a 1,1-glycosidic bond. An insect receives glucose with food, while trehalose is synthesized in its fat body and circulates in the hemolymph to be consumed by the insect’s muscle tissues [19]. While glucose’s function is conservative, and it serves as the primary source of energy in both flies and mammals, trehalose is the main source of energy insects use to fly. The genetic control of carbohydrate and lipid metabolism is a complex issue; however, in *D. melanogaster* several key genes participating in it have been identified. Thus, the *tps1* gene encodes trehalose-6-phosphate synthase converting glucose into trehalose, and the *treh* gene encodes trehalase, launching the reverse conversion. Insulin/insulin-like growth factor signaling (IIS) pathway also plays an important role in the control. In *Drosophila,* there is only one receptor; the gene (*dInR*) expression of which responds not only to feeding changes, but also to different adverse factors such as oxidative and heat stress [20,21]. As for lipid storage and triglyceride (TAG) homeostasis, in *Drosophila* they are controlled by the *bmm* gene, which encodes the lipid storage droplet-associated TAG lipase Brummer, a homolog of human adipocyte triglyceride lipase [22]. 

Recent studies have demonstrated that *Wolbachia* can affect the energy metabolism of their host and depend on glycolytic intermediates for energy supply [23]. Specifically, the bacteria seem to have to depend on intermediate carbohydrates to produce ATP and modulate the host’s metabolism in a way to obtain these molecules. Since *Wolbachia* are maternally inherited, this modulation probably starts during embryogenesis and continues throughout the host’s life [24]. Ponton et al. [25] demonstrated that *Wolbachia* changed the protein/carbohydrate ratio throughout *D. melanogaster’s* life. As a result, both infected and uninfected flies fed with proteins and carbohydrates in proportion 1:16 lived longer (pairwise comparisons, *p* = 0.125, median lifespan (mean ± s.e.): *Wolbachia*-infected = 27.1 ± 0.6 days, non-infected = 24.2 ± 0.6 days) than those fed in proportion 1:1; however, the latter, if uninfected, lived longer than infected ones (pairwise comparisons, *p* < 0.001, median lifespan (mean ± s.e.): *Wolbachia*-infected = 7.6 ± 0.6 days, non-infected = 8.2 ± 0.7 days) [25]. This phenomenon can probably be explained by a competition for carbohydrates between *Wolbachia* and the host, which results in a decrease in the lifespan of infected flies under carbohydrate deficiency conditions. 

To study the effect different *Wolbachia* genotypes have on *D. melanogaster* survivability, eight conplastic lines bearing isogenic wild type Bi90 line’s nuclear background and infected with different wMel and wMelCS *Wolbachia* genotypes were bred and characterized for their effect on *D. melanogaster* dopamine metabolism [26,27]. Here we investigate the effect these *Wolbachia* strains have on the carbohydrate and lipid metabolism and feeding behavior in *D. melanogaster* females. We believe this study will help clarify the physiological mechanisms underlying *Wolbachia’s* effect on host adaptation.

## 2. Materials and Methods

### 2.1. Drosophila Lines

To examine *Wolbachia’s* effect on *D. melanogaster* metabolism, eight lines with the nuclear background of wild type line Bi90 and different cytoplasmic backgrounds were used. Bi90 line was established from a wild-caught female of the Bishkek (Kyrgyzstan) population and interbred for more than 300 generations; thereby, it could be considered a nearly isogenic line. It was originally infected with wMel genotype of *Wolbachia*. One pair of flies from line Bi90 was isolated to create a branch, which was then treated with tetracycline for three generations to make *Wolbachia*-free line Bi90^T^. The six *D. melanogaster* conplastic lines carrying the nuclear background of Bi90 line and cytoplasm with different types of *Wolbachia* infection were created (Table 1) by 20 successive backcrosses of Bi90^T^ males with the appropriate source of *Wolbachia*, as described in [26,27]. *Wolbachia* donor lines were isolated from nature, maintained in the laboratory and characterized for infection [28,29,30]. *Wolbachia* infection status was regularly verified using PCR with primers specific to *Wolbachia*: the 81F/691R set for the wsp gene [31] and 99F/994R for the 16SrRNA gene [32]. The uninfected Bi90^T^ line was used as a control.

All lines were kept on standard *Drosophila* medium (agar–agar, 7 g L^−1^; corn grits, 50 g L^−1^; dry yeast, 18 g L^−1^; sugar, 40 g L^−1^) at 25 °C and relative humidity 50% under a 12 h:12 h light:dark cycle. Imagoes were synchronized at eclosion (flies were collected every 3–4 h). All the experiments were carried out on 6-day-old females.

### 2.2. Body Mass and TAG Content Measurements

For body mass evaluation, females from the control and experimental groups (10 flies per group, 1 fly at a time) were weighed using precision scales (Ohaus Corp. Pine Brook, Parsippany-Troy Hills, NJ, USA). TAG content was measured using Mukherjee and Mishra’s spectrophotometric assay [33]. Flies were grouped by 10 individuals to obtain a sufficient amount of TAGs per sample (3 samples were measured for each group under study). Flies were decapitated to avoid the effect of red pigment on the spectrophotometry, homogenized in 100 µL of a cooled PBST (0.2% Tween-20 (Medigen, Novosibirsk, Russia) в 1X PBS, (Invitrogen Corporation, Waltham, MA, USA)) and centrifuged for 5 min at 3075× *g*. Supernatant was transferred into a microcentrifuge tube, which was then heated at 70 °C for 10 min. Next, 20 μL of PBST or 20 μL of Triglyceride reagent (Sigma-T2449, St. Louis, MO, USA) was added to 20 μL of supernatant, PBST blank or glycerol standard. The tubes were incubated at 37 °C for 1 h and then centrifuged at 17.709× *g* for 3 min. A total of 30 μL from each tube were taken to a 96-well plate, 100 μL Free glycerol reagent (Sigma-F6428, St. Louis, MO, USA) was added to each well, and the plate was incubated at 37 °C for 5 min. The absorbance was measured at the wavelength of 540 nm using a Multiscan SkyHigh spectrophotometer (Thermo Fisher Scientific, Waltham, MA, USA). To evaluate the TAG concentration, the absorbance of free glycerol in the untreated sample without Triglyceride reagent was subtracted from the concentration of total glycerol of samples treated with Triglyceride reagent.

### 2.3. Colorimetric Method for Quantitative Determination of Total Lipid Content Using Sulfophosphovaniline (SPV) Reaction

Total lipid content was evaluated using modified Van Handel’s method [34]. For each experimental group, 9 to 14 flies were homogenized separately in 100 µL of a cooled mixture of chloroform–methanol (*v*/*v*) per fly. A total of 50 µL of the supernatant was shaken for 10 min, transferred into a clean test tube and heated at 90 °C in an M-208 microthermostat (Bis-N, Novosibirsk, Russia) until complete evaporation of the solvent. Next, 10 µL of H_2_SO_4_ was added to the samples, they were heated at the same temperature for 2 more min and then cooled on ice. Phosphovanillin reagent was added to the samples to a total volume of 1 mL and they were incubated at room temperature in the dark until a pink color appeared. The samples were then analyzed at a wavelength of 525 nm against a “blank” sample containing only Phosphovaniline reagent with the use of Smart Spec Plus spectrophotometer (Bio-Rad, Philadelphia, PA, USA).

### 2.4. Spectrophotometric Method of Evaluation of Carbohydrates Metabolism

The glucose titer in *D. melanogaster* females was evaluated with the use of a Glucose (HK) Assay Kit (Lot #SLBL3912V; Sigma-Aldrich, St. Louis, MO, USA) using the spectrophotometric method of Musselman et al. [35] with adjustments. Flies were decapitated to eliminate eye pigment, homogenized in a hypotonic lysing buffer (20 mM HEPES, 2 mM MgCl_2_, 2 mM EGTA) and then placed into a cooled microcentrifuge tubes. After 10 min of incubation, samples were centrifuged at 13,400× *g* for 5 min and titers of metabolites were determined in supernatant. To evaluate trehalose titer, it was converted into glucose by adding trehalase (Sigma-Aldrich; 0.5 U/mL) with a further measurement of glucose concentration. The samples were analyzed at a wavelength of 340 nm with the use of a Smart Spec Plus spectrophotometer (Bio-Rad, Philadelphia, PA, USA). Each experiment was performed with three biological replicates.

### 2.5. Quantitative Real-Time PCR (qPCR)

The relative mRNA amount was evaluated using qRT-PCR. The total RNA was extracted from whole bodies of 6-day-old *D. melanogaster* females (25 flies per sample for every biological replicate) using TRI reagent (Lot #BCBT8883, Sigma-Aldrich, St. Louis, MO, USA). The remaining genomic DNA was removed from the samples by processing them with DNase I (Promega Corporation, Madison, WI, USA) according to the manufacturer’s instructions. The complementary DNA was synthesized from 1 µg of total RNA using a RevertAid First Strand cDNA Synthesis Kit (Thermo Fisher Scientific, Vilnius, Lithuania, USA) with an oligo(dT)18 primer as per the manufacturer’s protocol. Primers were synthesized by Biolabmix (Novosibirsk, Russia).

qRT-PCR was carried out in a reaction mixture of 20 µL in the presence of SYBR-Green I (Syntol, Moscow, Russia) in a CFX96 Real Time System amplifier (Bio-Rad, Hercules, California, USA) under the following conditions: initial 3 min denaturation at 95 °C followed by 45-cycle amplification, each cycle took 15 s at 95 °C; 15 s annealing at 56–62 °C (depending on primers in use); cyclic amplification followed by 5 s elongation at 78 °C; the melting curve covered a temperature range from 65 to 95 °C. The relative mRNA amount to determine the expression levels of the *tps1* (F: CGTGTGACATCGTCGGATATT, R: AGTGTCGTTCCACCCATTTC), *treh* (F: GAAAATGCTGTCTCTCTCGCTC, R: ATTCCTGGCGGTGCTGTA), *bmm* (F: AATGGCGTCGAATCAGACTT, R: AACACAGATGGGGATTTGGA) and *dInR* (F: AAGCGTGGGAAAATTAAGATGGA, R: GGCTGTCAACTGCTTCTACTG) genes was calculated by applying the comparative Ct method [36] using Actin 5C (F: GCGCCCTTACTCTTTCACCA, R: ATGTCACGGACGATTTCACG) and β-Tubulin (F: TGTCGCGTGTGAAACACTTC, R: AGCAGGCGTTTCCAATCTG) as reference genes regularly expressed in 6-day-old females infected with different *Wolbachia* strains. Each reaction was performed in triplicate with three biological replicates. 

### 2.6. Capillary Feeding Assay

The food consumption was evaluated as in Williams et al. [37]. A total of 5 mated 6-day-old females of each *D. melanogaster* line under study were placed in a vial, 10 cm × 2 cm (height × diameter), which contained 1% agarose (5 cm high) providing moisture and humidity for the flies during the experiment. A capillary glass tube (10 × 80 mm) was filled with 15 μL of liquid food (5% sucrose, 5% yeast extract, 90% water) and was put in a vial stopper using two pipet tips (plugged into one another). The initial food level in the capillary tube was marked and 0.1 μL of mineral oil was applied over the liquid food to prevent its evaporation. The vials were kept in a 25 °C incubator for 24 h, and then the total food intake per day was evaluated by marking the final food level in the capillary tube. The “blank” vial without flies was used to detect the rate of food evaporation. The average food consumption was calculated by dividing the total food intake (minus the “blank” value) by the number of flies in the vial (5 vials for each line). Each experiment was performed with four biological replicates.

### 2.7. Starvation Resistance Analysis

For starvation resistance analysis, 5 6-day-old females were placed into a vial (10 vials per group) with pure sugar medium (20 g of agar and 4 g of sucrose per 1 L of water) and then were transferred to vials with fresh medium daily. The daily survival rate (DSR) was calculated every day as a proportion of flies alive per day to a total number of flies enlisted in the experimental group initially.

### 2.8. Statistical Analysis

Data were analyzed via the Kruskal–Wallis ANOVA (with infection as the between-subjects factor). The data on starvation resistance were analyzed via one-way ANOVA (infection as a factor). Differences between groups were assessed using Dunn’s post hoc, in which *p* ≤ 0.001 was considered highly significant and *p* ≤ 0.05 was considered significant.

## 3. Results

### 3.1. Lipid Content

**Figure 1:**
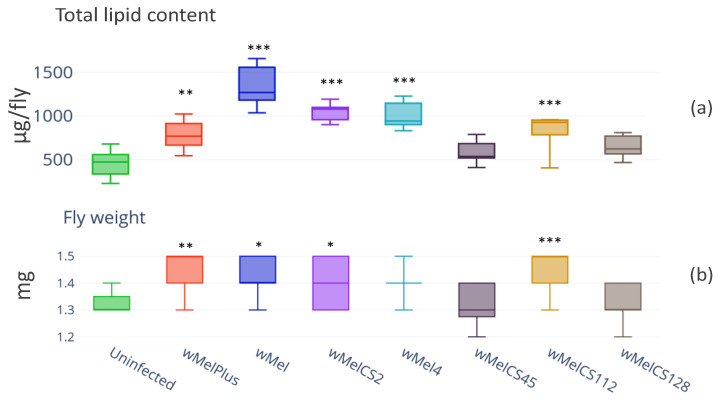
Total lipid content (**a**) and weight (**b**) in wild type Bi90 *D. melanogaster* females infected with wMel, wMel4, wMelPlus, wMelCS2, wMelCS45, wMelCS112 and wMelCS128 *Wolbachia* strains compared to that in uninfected Bi90^T^ control line. The asterisk indicates significant differences (*** *p* < 0.001, ** *p* < 0.01, * *p* < 0.05). A total of 8–14 measurements were made for each group.

The total lipid content was investigated in uninfected flies and the flies infected with *Wolbachia* of wMel (wMel and wMel4) and wMelCS (wMelPlus, wMelCS2, wMelCS45, wMelCS112 and wMelCS128) genotypes as it was shown earlier that one of the strains from wMelCS group, wMelPlus, had a positive effect on the host’s heat stress tolerance [27]. It was found that the lipid content was higher in the infected lines compared to that of the control (Figure 1a, infection—H_(7.67)_ = 71.7, *p* = 6.69 × 10^−13^). For lines Bi90^wMel^, Bi90^wMelCS2^, Bi90^wMel4^ and Bi90^wMelCS112^, the statistical difference with the control was significant at *p* < 0.001; for Bi90^wMelPlus^, *p* < 0.01. Evidently, *Wolbachia* facilitated the increase in the lipid stores of the host. 

The weights measured in lines Bi90^wMel^, Bi90^wMel4^, Bi90^wMelPlus^, Bi90^wMelCS2^, Bi90^wMelCS45^, Bi90wMelCS112 and Bi90^wMelCS128^ (Figure 1b) were matched against the lipid contents obtained for these lines and were higher than in the control (H_(7.67)_ = 71.7, *p* = 6.69 × 10^−13^). For line Bi90^wMelCS112^, the statistical difference with the control was significant at *p* < 0.001; for Bi90^wMel^, Bi90^wMelPlus^ and Bi90^wMelCS2^, *p* < 0.01, and for Bi90^wMel4^ differences were on the verge of significance (*p* = 0.089). 

At the same time, the total lipid content and weight of the flies of Bi90^wMelCS45^ and Bi90^wMelCS128^ lines did not differ from the control Bi90^T^ ones. 

The obtained results enabled us to choose three *Wolbachia* strains (wMel, wMelPlus and wMelCS45) that had the most contrasting effects on their host’s lipid content for further analysis. 

**Figure 2:**
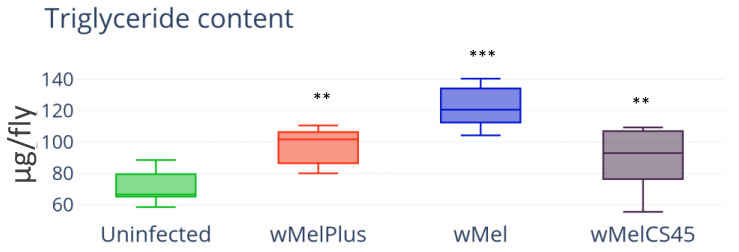
TAG content in wild type line Bi90 females infected with wMel, wMelPlus and wMelCS42 *Wolbachia* strains compared to that in uninfected Bi90^T^ control line. The asterisk indicates significant differences (*** *p* < 0.001, ** *p* < 0.01). Each experiment was performed with three biological replicates.

First, the amount of TAGs [38], the main storage fraction of lipids in *Drosophila*, was measured in the control and the three infected lines (Figure 2). The measurement results demonstrated that TAG content was statistically higher in all studied infected lines compared to the control (infection—H_(4.30)_ = 37.44, *p* = 3.86 × 10^−6^) including line Bi90^wMelCS45^, which did not differ from the control in total lipid content and weight. The statistical difference with the control was significant at *p* < 0.001 for Bi90^wMel^; it was at *p* < 0.01 for Bi90^wMel45^ and Bi90^wMelPlus^. This allowed us to assume that any *Wolbachia* strain might have an effect on its host’s energy metabolism. 

To test this assumption, we examined the carbohydrate content in the same three infected lines, since carbohydrates, like lipids, are essential for proper energy exchange. 

### 3.2. Carbohydrate Content

**Figure 3:**
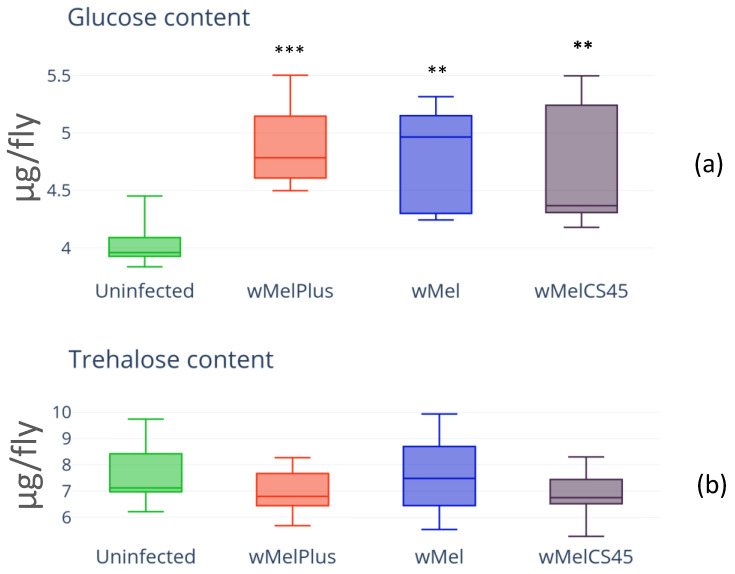
Glucose (**a**) and trehalose (**b**) content in females of *D. melanogaster* wild type Bi90 line infected with wMel, wMelPlus and wMelCS42 *Wolbachia* strains compared to that in uninfected Bi90^T^ control line. The asterisk indicates significant differences (*** *p* < 0.001, ** *p* < 0.01). Each experiment was performed with three biological replicates.

The content of two main insect carbohydrates, trehalose and glucose, was measured in females of the infected lines Bi90^wMel^, Bi90^wMel45^ and Bi90^wMelPlus^ and compared to that of the control line Bi90^T^. The results are presented in Figure 3. 

The measurement results showed increased glucose content, as well as TAG content, in all infected lines regardless of *Wolbachia* genotype if compared to the control (Figure 3a, H_(3.30)_ = 27.22, *p* = 1.79 × 10^−5^). Notably, the trehalose level remained unchanged in all infected lines (Figure 3b, H_(3.44)_ = 2.861, *p* = 0.4136). 

### 3.3. Evaluation of the Effect the Wolbachia Infection Had on the Expression of Key Genes for Carbohydrate and Lipid Metabolism Using Quantitative Real-Time PCR

**Figure 4:**
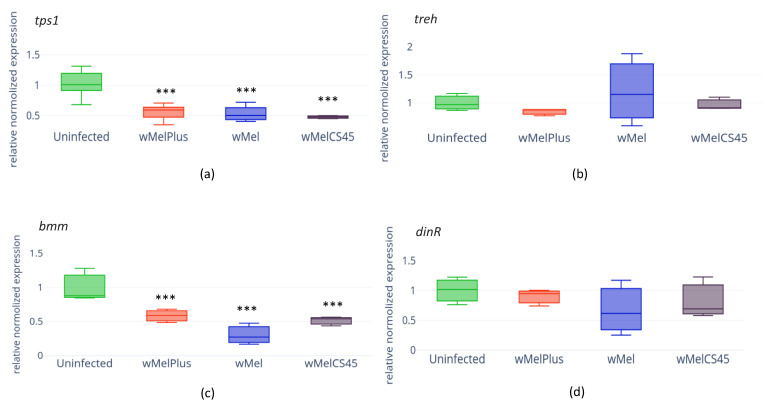
*tps1* (**a**), *treh* (**b**), *bmm* (**c**) and *dInR* (**d**) gene expression intensity in wild type Bi90 *D. melanogaster* females infected with wMel, wMelPlus and wMelCS42 *Wolbachia* strains compared to that in uninfected Bi90^T^ control line. The asterisk indicates significant differences (*** *p* < 0.001). Each experiment was performed with three biological replicates.

In the search for mechanism providing the changes in carbohydrates’ and TAG contents that we found, we performed qRT-PCR analysis of expression of several genes involved in carbohydrate and lipid metabolism. We demonstrated that any *Wolbachia* strain reduced the expression of the *tps1* gene (coding for an enzyme for trehalose synthesis from glucose) but did not affect the expression of the *treh* gene (coding for an enzyme that degrades trehalose to produce glucose) in all infected lines (Figure 4a, infection—H_(3.8)_ = 9.435, *p* = 0.02 for *tps1*; and Figure 4b, infection—H_(3.8)_ = 0.598, *p* = 0.47 for *treh*). The activity of the *bmm* gene encoding for the homolog of human adipocyte triglyceride lipase and playing a key role in regulation of lipid metabolism in *D. melanogaster* was demonstrated to be reduced in all infected lines (Figure 4c, infection—H_(3.8)_ = 9.154, *p* = 0.02). 

To estimate IIS condition in the infected flies, the level of *dInR* expression was measured, and the results demonstrated that none of the endosymbiont strains under study affected it (Figure 4d, infection—H(3.8) = 1.932, *p* = 0.58).

### 3.4. Feeding Behavior and Starvation Resistance

**Figure 5:**
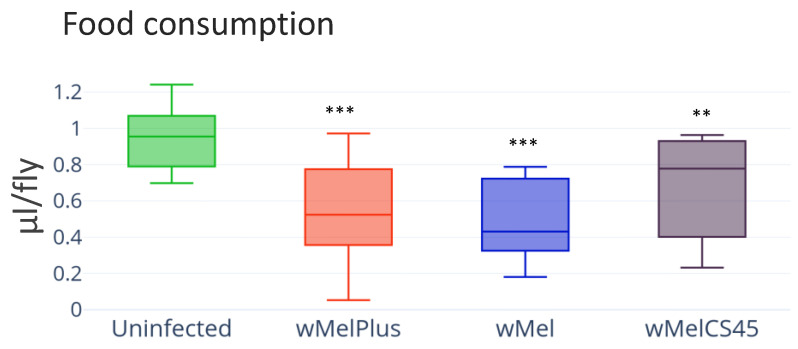
Analysis of food consumption in wild type line Bi90 females infected with wMel, wMelPlus and wMelCS42 *Wolbachia* strains compared to that in uninfected Bi90^T^ control line. The asterisk indicates significant differences (*** *p* < 0.001, ** *p* < 0.01). A total of 11–12 measurements were made for each group.

Based on the detected changes in carbohydrate and lipid contents in the *Wolbachia*-infected lines, an assumption was made that differences would also be seen in the flies’ feeding behavior. To clarify this issue, the effect of wMel, wMelPlus and wMelCS45 strains on *D. melanogaster* feeding behavior was estimated. For this purpose, the intensity of 24 h capillary feeding of 6-day-old females was assessed (Figure 5). The measurements demonstrated a statistically significant decrease in the food consumption in all the infected lines compared to the control (infection—H_(5.54)_ = 12.56, *p* = 0.02), which was a highly unexpected result.

**Figure 6:**
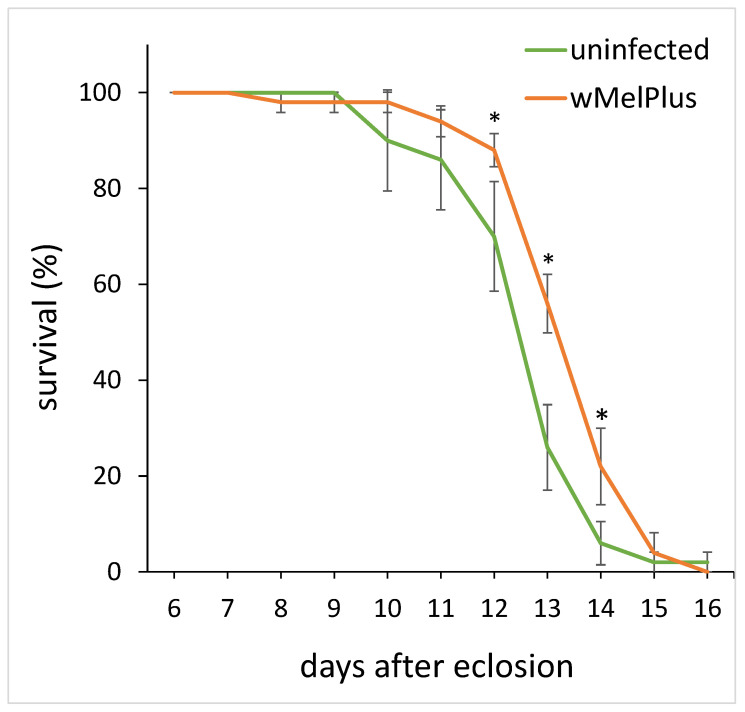
The daily survival rate in females of uninfected wild type line Bi90 and Bi90 line infected with wMelPlus *Wolbachia* strain placed on pure sugar medium on the 7th day after eclosion. The asterisk indicates significant differences (*p* < 0.05). Each group under study includes 50 females (10 vials, 5 females per vial).

However, in spite of the decreased appetite of the females infected with wMelPlus *Wolbachia* strain, their survival under starvation turned out to be higher than that of the females of uninfected Bi90^T^ control line (Figure 6).

## 4. Discussion

The performed investigation demonstrated that *Wolbachia* stimulated carbohydrate metabolism in *D. melanogaster,* which was confirmed by a higher level of glucose in the infected lines than in the control. Since it became an intercellular endosymbiont, *Wolbachia* have lost many of their genes, including those responsible for metabolism [39]. Thus, it stands to reason that the bacteria rely on their host for providing components necessary for survival and reproduction. This idea has also been supported by other authors, e.g., Zhang et al. [40] found that the glucose level in *Wolbachia*-infected male flies significantly exceeded that in non-infected ones. Using comparative metabolomics analysis, they also discovered that the transcription level of the glycolysis-related genes such as *Gapdh* (coding for the glyceraldehyde-3-phosphate dehydrogenase) and *Adh* (coding for the alcohol dehydrogenase) turned out to be significantly higher due to *Wolbachia* infection. Their study also demonstrated a significant reduction of glycogen (main storage sugar) content in *Wolbachia-*infected *D. melanogaster* males compared to an uninfected control. The increased glucose content found in our study probably occurred for the same reason. Increased levels of glucose-6-phosphate have also been described in *Wolbachia*-infected mosquitoes *A. fluviatilis* [41].

In the case of trehalose, no *Wolbachia*-related effect was found, and the level of this carbohydrate was similar in both infected and uninfected flies, which was probably due to glucose regulation being more dynamic compared to trehalose. That had been demonstrated earlier for *D. melanogaster* larvae [42]. Ugrankar et al. [43] also demonstrated that in different physiological conditions, including breeding in crowded conditions, in larvae, the level of circulating glucose, but not of trehalose, was regulated. In *D. melanogaster*, trehalose is synthesized in its fat body from glucose through the catalytic activity of the Tps1 protein [44], and losing Tps1 results in trehalose deficiency in animals [45]. Trehalose-deficient larvae are sensitive to nutrient shortage and quickly die during starvation [44]. The trehalase encoding by *treh* gene catalyzes trehalose hydrolysis to produce glucose and Treh’s inactivation prevents trehalose catabolism, significantly increasing its content in hemolymph [44]. The data obtained in our study demonstrate that *Wolbachia* reduced *tps1* gene expression and had no effect on the *treh* gene, which is probably why the trehalose level remained the same at the high levels of glucose observed in the infected flies.

The detected effect *Wolbachia* had on the level of *dInR* expression corresponds with the unchanged trehalose level in the infected flies since IIS regulation of this carbohydrate has been found in *D. melanogaster* and *Bombyx mori* [46,47,48,49,50,51]. Thus, in *B. mori* the IIS affects trehalose homeostasis through the expression of the *treh* gene [47,48].

**Figure 7:**
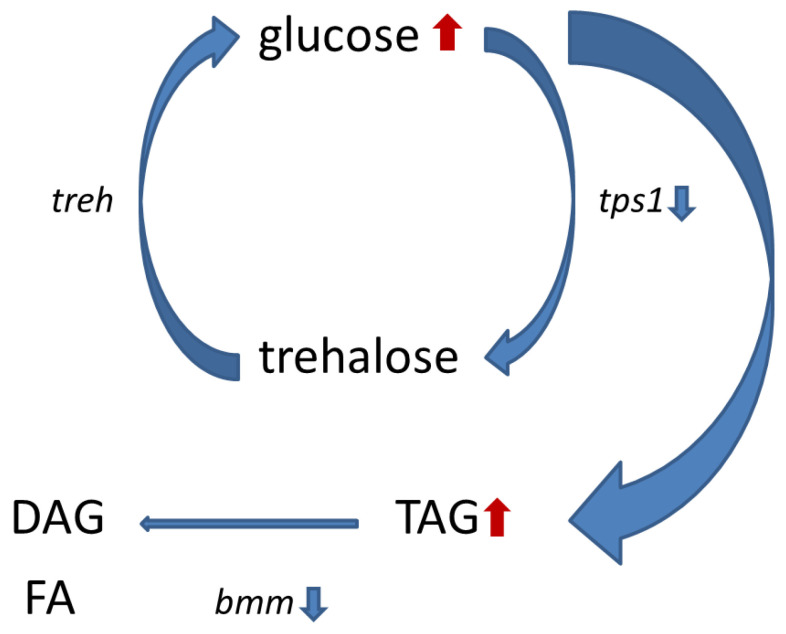
Scheme of *Wolbachia* effect on the regulation of carbohydrate and lipid metabolism in *Drosophila*, where TAG—triglycerides, DAG—diglycerides, FA—fatty acids, *tps1—*gene coding for Trehalose-6-phosphate synthase 1, the enzyme synthesizing trehalose from glucose, *treh—*gene coding for trehalase, the enzyme of trehalose degradation, *bmm—*gene regulating TAG metabolism.

*Wolbachia* also do not have the key genes for lipid biosynthesis, so they probably rely on their host in this respect as well [52]. That is why the infection significantly increased lipid content in the studied infected lines, increasing the total lipid level and that of TAG, which corresponds to the data published by Zhang et al. [40] who demonstrated that *Wolbachia*-infected *Drosophila* males had higher levels of linolenoyl-CoA, palmitoleic and gamolenic acids involved in lipid metabolic pathway, TAGs and many other intermediate products of fatty acid metabolism compared to those of the control. In the experiments with *A. aegypti* artificially infected with *w*Mel *Wolbachia*, reduced expression of the *bmm* gene, playing the key role in the *Drosophila* lipid metabolism regulation, was found in all the infected lines investigated [53]. Taken together, this implies that *Wolbachia* decreases lipolysis through the expression inhibition of certain genes, reducing the activity of the enzymes enabling TAG degradation. The scheme of a supposed *Wolbachia* effect on the regulation of carbohydrate and lipid metabolism in *D. melanogaster* is shown in Figure 7.

As lipids deposited into the oocytes significantly contribute to the whole body fat level, and oocytes are one of the main locations of *Wolbachia* in *Drosophila’s* tissues, further studies in this field may include an investigation of potential changes in oogenesis input in the obese phenotype of *Wolbachia*-infected flies.

Reduced appetite in the infected flies in our study came as a surprise since increased starvation resistance and glucose’s and TAGs’ levels allowed an assumption of an intensification of food intake. However, the data published on this subject are rather contradictory. Turley at al. [54] investigated the feeding behavior in the mosquito and found that transinfecting *A. aegypti* with *Wolbachia* strain wMelPop reduced the frequency and volume of blood consumption by females. The experiments performed by Zhang et al. [40] in *D. melanogaster* males demonstrated that the *Wolbachia* infection led to a significant increase in consumed food during 24 h after eclosion. He et al. [55] compared the feeding behavior of *D. melanogaster* females after coupling with *Wolbachia*-infected and uninfected males and observed that the females coupling with infected males had a higher feeding frequency if compared to those coupling with uninfected males. At the same time, the infection status of the females did not affect the feeding frequency, which implies the infected males could cause a significant reduction in the amount of food consumed by their females. This corresponds to our data, since the females used in our experiments coupled with the males of the same *Wolbachia* infection status. Possibly, the enhanced metabolism of infected insects enables them to reduce their need for nutrients for a while, whereas at other times, they can accumulate nutrients in double the amount, which increases the survivability of *Wolbachia*-infected insects under unfavorable environmental conditions. This suggestion allows one to explain the wide spread of *Wolbachia* in *D. melanogaster* populations despite the mild cytoplasmic incompatibility in this species [56]. Cytoplasmic incompatibility occurs when *Wolbachia*-infected males mate with uninfected females, which results in embryo death; this is considered one of the most significant mechanisms behind the spread of *Wolbachia* in host populations.

## 5. Conclusions

It can be assumed that in *D. melanogaster*, *Wolbachia* transform the host’s metabolism in different ways to affect the energy exchange and starvation resistance, providing the host with a competitive advantage which increases the infection’s spread in the population.

## Figures and Tables

**Table 1:** *Drosophila* lines used in the study.

*Drosophila* Line	*Wolbachia* Infection	Line-Donor of Cytoplasm	Origin of Donor Line
Bi90^T^	---	Bi90, tetracycline treated	Kyrgyzstan, 2004
Bi90^wMel^	wMel	Bi90	Kyrgyzstan, 2004
Bi90^wMelPlus^	wMelPlus	w153	Uzbekistan, 1989
Bi90^wMelCS128^	wMelCS	1-128	Australia, 1986
Bi90^wMelCS45^	wMelCS	45	Sankt-Peterburg, Russia, 1995
Bi90^wMelCS112^	wMelCS	3-112	Bloomington, USA, 2010
Bi90^wMelCS2^	wMelCS2	93220	Biysk, Altai, Russia, 1993
Bi90^wMel4^	wMel4	w304	Sinai Peninsula, Egypt, 2010

## Data Availability

The data is available within this article.
